# Tau *N*-Terminal Inserts Regulate Tau Liquid-Liquid Phase Separation and Condensates Maturation in a Neuronal Cell Model

**DOI:** 10.3390/ijms22189728

**Published:** 2021-09-08

**Authors:** Chengchen Wu, Junyi Zhao, Qiuping Wu, Qiulong Tan, Qiong Liu, Shifeng Xiao

**Affiliations:** 1 Shenzhen Key Laboratory of Marine Biotechnology and Ecology, College of Life Sciences and Oceanography, Shenzhen University, Shenzhen 518060, China; katharinewu2007@hotmail.com (C.W.); 1800251011@email.szu.edu.cn (J.Z.); wuqiuping2017@email.szu.edu.cn (Q.W.); tanqiulong@szu.edu.cn (Q.T.); liuqiong@szu.edu.cn (Q.L.); 2 Shenzhen-Hong Kong Institute of Brain Science-Shenzhen Fundamental Research Institutions, Shenzhen 518055, China

**Keywords:** liquid–liquid phase separation, tau isoforms, protein aggregation, p62 protein

## Abstract

The microtubule-associated protein tau can undergo liquid–liquid phase separation (LLPS) to form membraneless condensates in neurons, yet the underlying molecular mechanisms and functions of tau LLPS and tau droplets remain to be elucidated. The human brain contains mainly 6 tau isoforms with different numbers of microtubule-binding repeats (3R, 4R) and *N*-terminal inserts (0*N*, 1*N*, 2*N*). However, little is known about the role of *N*-terminal inserts. Here we observed the dynamics of three tau isoforms with different *N*-terminal inserts in live neuronal cell line HT22. We validated tau LLPS in cytoplasm and found that 2*N*-tau forms liquid-like, hollow-shell droplets. Tau condensates became smaller in 1*N*-tau comparing with 2*N*-tau, while no obvious tau accumulated dots were shown in 0*N*-tau. The absence of *N*-terminal inserts significantly affected condensate colocalization of tau and p62. The results reveal insights into the tau LLPS assembly mechanism and functional effects of *N*-terminal inserts in tau.

## 1. Introduction

Liquid–liquid phase separation (LLPS) has been observed within the cells at the molecular level to allow membraneless compartmentalization and regulation of biological processes, such as gene expression, autophagic degradation, assembly of signaling clusters, formation of synaptic density signaling assemblies, asymmetric segregations of cell fate determinants, etc. [1,2,3,4] However, the assembly and aggregation mechanisms of the biomolecular interactions giving rise to phase separation and the functions of such biomolecular condensates are still elusive. Low complexity domains [5] and intrinsically disordered regions [6] have been almost invariantly found in proteins that can undergo LLPS and form biomolecular condensates, as either could work as the initial driving force for biomolecules phase separation.

In neurons, the intrinsically disordered microtubule-associated protein tau has been reported to efficiently undergo LLPS to form condensed liquid droplets [7], which are clearly distinct from fibrillary aggregates in neurofibrillary tangles in neurodegenerative diseases [8]. Nevertheless, the hypothesis has been proposed that LLPS-driven tau droplet formation may represent a common and critical mechanism as the initial step to further lead to tauopathies [7], a group of progressive neurodegenerative disorders that are pathologically defined by the presence of tau protein aggregates in the brain [9]. The canonical role of tau protein is to stabilize and bundle axonal microtubules in healthy neurons [10], and it exists in six predominant isoforms in the human brain that result from alternative splicing of exons E2, E3, and E10, giving rise to the variants containing either 0, 1, or 2 *N*-terminal inserts (0*N*, 1*N*, and 2*N*), and 3 or 4 microtubule-binding repeats (MTBR) (3R and 4R) [11]. Tau LLPS is regulated by intermolecular electrostatic interactions [12] and post-translational modifications, such as phosphorylation [7,13] and acetylation [14]. The RNA and RNA binding protein TIA1 can also interact with tau to drive tau LLPS [15]. The *C*-terminal microtubule-binding repeats of tau are the aggregation-prone region [16], which are believed to facilitate tau aggregation and play a crucial role in the formation of neurofibrillary tangles in Alzheimer’s disease (AD). They can also undergo LLPS in vitro, while three-repeat (3R) and four-repeat (4R) isoforms of tau differ in their ability to produce LLPS [13]. Moreover, the proline-rich domain (PRD) is also shown to promote tau LLPS, and, interestingly, tau PRD forms heterotypic condensates with EB1, a regulator of microtubule dynamic instability [17]. However, the *N*-terminal inserts of tau are less studied, and their roles and functions in forming tau LLPS still need to be explored. Besides, while in vitro tau LLPS studies on triggering by crowding agents, such as PEG and heparin, have been reported [8,18,19], definitive evidence is lacking as to whether the same principles apply in the biological environment inside living cells [20]. Biomolecular condensate formation and dynamics in living cells with proper temperature and pH simulate more complicated biological environments and sophisticated biochemical processes, which more proximately mimic biomolecular dynamics in living organisms.

In our study, we constructed three stably-transfected tau isoforms with 0, 1, or 2 *N*-terminal inserts in neuronal cell line HT22 to investigate the contribution of *N*-terminal inserts in tau LLPS in cultured cells. We validated tau LLPS in real-time live cells and observed that droplet-like tau condensate may have a hollow-shell structure and that the *N*-terminal inserts of tau play a critical role in LLPS-driven tau condensate’s formation and maturation. Moreover, the deficiency of tau *N*-terminal inserts can significantly affect tau co-condensed with p62, an autophagy adaptor protein that can undergo LLPS and shuttle ubiquitinated cargo for autophagic degradation [21]. Generally, our findings reveal the role of tau *N*-terminal inserts in tau LLPS in live neuronal cell lines, and in the context of the previously proposed hypothesis that tau droplet (spherical droplet-shaped accumulations) formation can lead to tau aggregation [7], tau *N*-terminal inserts may hold promise as a novel therapeutic target to intervene in tau pathological aggregation in neurodegenerative diseases.

## 2. Results

### 2.1. Tau Undergoes LLPS in Live Neuronal Cell Line HT22

**Figure 1:**
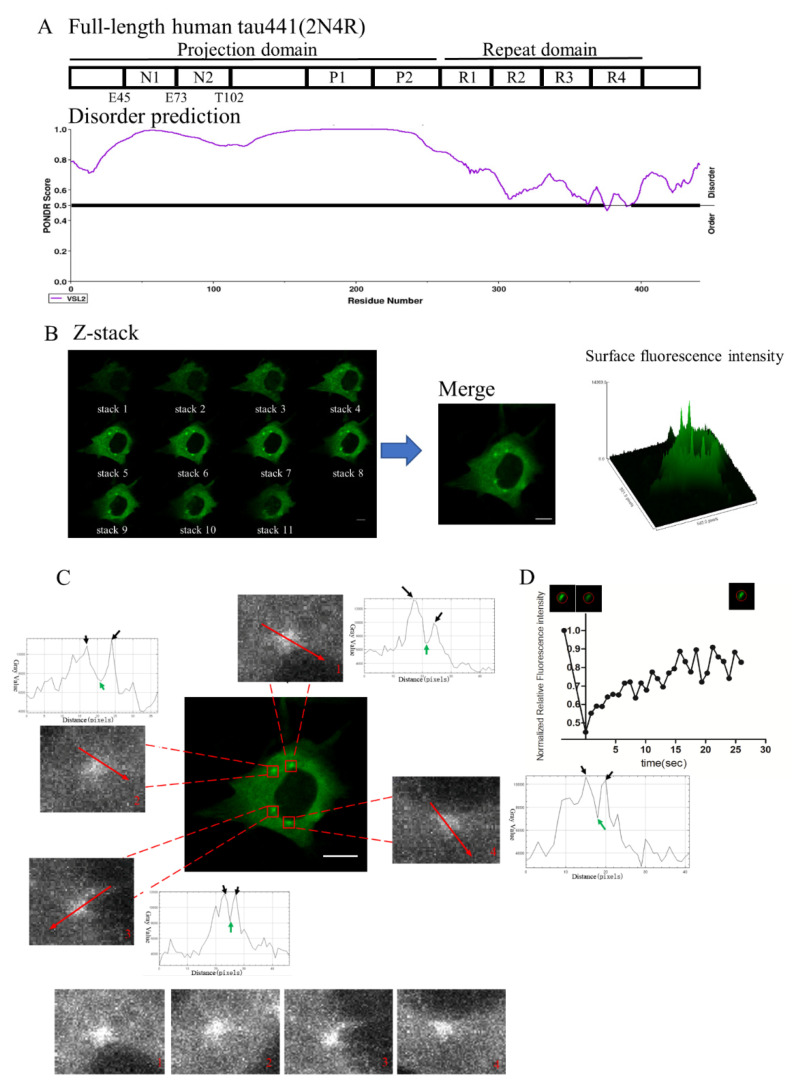
Tau undergoes LLPS in a neuronal cell line. (**A**) Protein sequence and disorder prediction of the 2*N*4R tau. (**B**) Intracellular droplet-like accumulation of tau-EGFP in live HT22. The graph shows the tau-EGFP fluorescence intensity in cytoplasma. Scale bar = 10 μm. (**C**) Cross-sectional profiles of tau-EGFP condensates. Red arrows represent the selected locations of cross-sections on tau droplets. In the cross-sectional profiles, black arrows represent the peak gray value, and green arrows represent the bottom gray value (gray value equals the fluorescent intensity of tau). The four unobscured images are displayed below. Scale bar = 10 μm. (**D**) FRAP of tau-EGFP condensates.

Tau is intrinsically disordered (Figure 1A), and the *N*-terminal inserts and PRD in the projection domain both display a high propensity to undergo LLPS. The full-length 2*N*4R-tau was inserted before the GFP tag in the pMSCV-GFP plasmid, and the protein complex was stably expressed with a retrovirus transfection in the HT22 murine hippocampal neuronal cell line. As shown in Figure 1B, intracellular droplet-like accumulations of tau-EGFP were observed in the cytosol of live HT22 cells. No droplets were observed in the cells transfected with GFP alone (data not shown), as consistent with both in vitro and in vivo studies [7,19]. Furthermore, we also explored the structure and organization of the tau condensates. Notably, the cross-sectional profiles of the EGFP signal revealed that tau proteins were not evenly distributed in the condensates. However, tau proteins were largely distributed on the shell because the peak fluorescent intensity constantly showed on the shell while being less condensed inside the tau droplet (Figure 1C). This may reveal that tau formed a core-shell spheroidal structure which was extensively observed in molecules undergoing LLPS [22]. Besides, the dispersed tau was filled and dense in cytoplasma (Figure 1B,C). The FRAP curve showed that the tau condensates partially fast recovered after photobleaching, with an immobile tau molecule fraction of about 10% (Figure 1D). Consistent with previous studies [7,12,18], this indicated fast tau protein diffusion and a dynamic exchange between tau condensates and dispersed tau in the cells, validating the liquid-like property of tau condensates. Thus, 2*N*-tau undergoes LLPS to form a liquid-like, hollow-shell structure in live HT22 cells.

### 2.2. Tau N-Terminal Inserts Regulate Tau Condensate Formation

**Figure 2:**
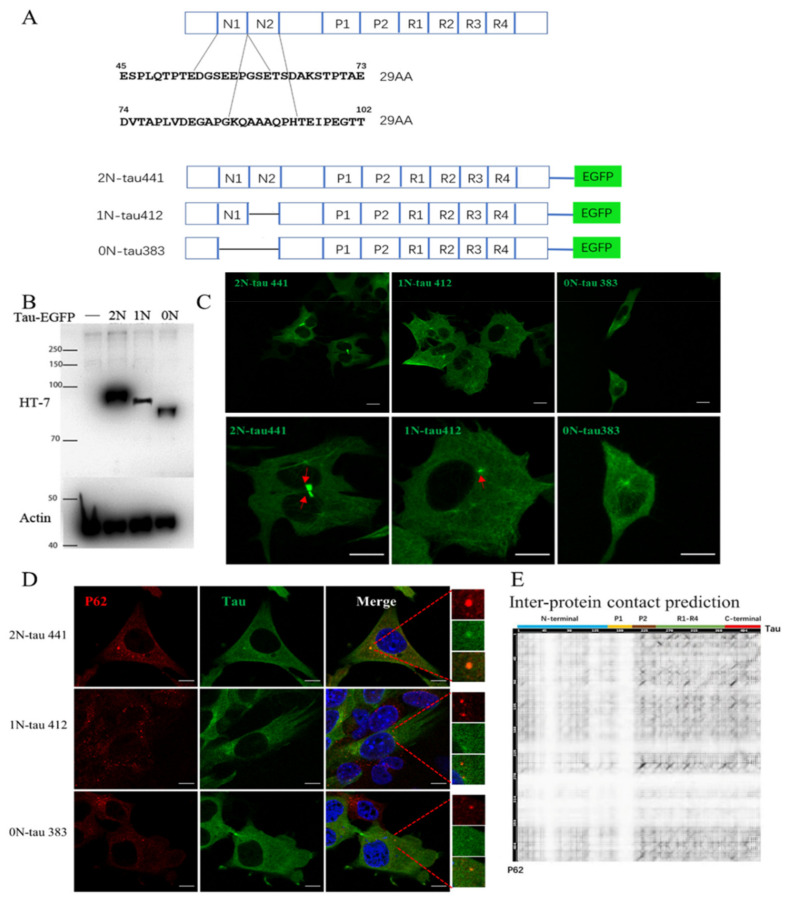
The *N*-terminal inserts are essential for tau condensate formation and maturation. (**A**) Schematic diagram of 3 human tau isoforms with different *N*-terminal inserts. (**B**) Western blotting of cell extracts from stable tau-EGFP transfected cells. The first lane represents the negative control. (**C**) Intracellular tau condensate accumulation in live HT22 with different *N*-terminal inserts of tau. Scale bar = 10 μm. (**D**) p62 inclusion selectively colocalizes with tau condensates. Scale bar = 10 μm. (**E**) Heatmap of the probabilities of residues involved in contact between tau and p62 using ComplexContact. The ordinate axis denotes p62, while the horizontal axis tau with annotation of the protein domains above. A higher probability is represented by the darker color.

To investigate the role of *N*-terminal inserts in tau LLPS formation in live cells, three tau isoforms with 0, 1, or 2 amino terminal inserts, respectively (0*N*, 1*N*, 2*N*-tau), were studied (Figure 2A). No obvious tau condensates could be observed with 0*N*-tau, while scattered tau droplets appeared using 1*N*-tau. Notably, using 2*N*-tau, the condensates appeared in most cells and were bigger in size compared to those in 1*N*-tau (Figure 2C). Additionally, tau was more outstretched in 2*N*-tau (Figure 2C). The live-cell imaging results suggest that the *N*-terminal inserts of tau play a critical role in tau droplet formation and growth, and the tau LLPS was dysregulated when *N*-terminal inserts of tau were absent. We propose that *N*-terminal inserts of tau are closely related to the biogenesis of tau LLPS-driven condensate formation in live HT22 cells.

### 2.3. Tau N-Terminal Inserts Regulate Tau Co-Condensation with p62

We postulated that the liquid-like, hollow-shell structure of 2*N*-tau may behave like a scaffold that anchors interacting biomolecules to accelerate or suppress biochemical reactions to perform specific biological functions in cells. To test this hypothesis, we investigated the co-condensation of biomolecules with tau droplets. Multiple coexisting liquid phases immiscible with one another were observed in cells [23]. The autophagy adaptor protein p62 was found to co-condensate with 2*N*-tau droplets, and, interestingly, tau droplets seemed to be encapsulated in p62 inclusion (Figure 2D). However, in 1*N*-tau, the colocalization could not be observed between tau condensates and p62 droplets. No obvious tau condensates were shown in 0*N*-tau, while several p62 droplets still appeared in cells. Moreover, the expression level of p62 and formation of p62 condensates in 0*N*-tau were much less compared to those in 2*N*-tau (Figure 2D). The scaffold protein p62 is a cargo receptor in selective autophagy [24] and has been shown to undergo phase separation to drive autophagic cargo concentration and segregation as well as, consequently, protein degradation by autophagosomes [25]. The deficiency of tau *N*-terminal inserts can significantly affect tau co-condensed with p62, suggesting the critical role of tau *N*-terminal inserts in tau protein degradation and quality control in cells. Furthermore, we also explored the protein interaction between tau and p62 in silico. The heatmap of residue contact prediction (Figure 2E) showed that the interfacial contact residues of tau to p62 were mostly distributed in P2, R1, R2 and *C*-terminal regions, indicating no direct contact between tau *N*-terminal inserts and p62. Thus, further exploration should focus on how *N*-terminal inserts of tau affect 2*N*-tau and p62 co-condensation when no direct contact is shown between *N*-terminal inserts of tau and p62.

## 3. Discussion

Tau protein dysfunction and accumulation is the most common pathology among over 20 degenerative brain diseases, including AD, progressive supranuclear palsy, traumatic brain injury, and others [26,27]. Neurodegenerative diseases are associated with aging. Emerging evidence supports the hypothesis that aberrant phase separation behavior may serve as a trigger for protein aggregation in neurodegeneration with time [28] and that tau isoforms gradually shift through the development in protein with time [29]. Different tau isoforms are reported to contribute to heterogeneous tauopathies [30]. The aggregation-prone repeat region of tau from the dispersed monomeric state to phase-separated liquid-like droplets involves the aggregation-prone hexapeptides (^275^VQIINK^280^ in R2 and ^306^VQIVYK^311^ in R3) and regulatory KXGS motif in *C*-terminal MTBR [16]. However, less is known about the initial step toward tau aggregation and tau pathology. Understanding the initiation and processing of dispersed monomeric tau toward progressively pathological aggregates may support the development of novel therapeutic approaches for tauopathies.

The *N*-terminal domain in tau has been described as flexible and inherently dynamic with a long, unstructured tail, while the proline-rich domain and *C*-terminus have been featured in a more ordered fashion, which is more likely to trigger tau LLPS. It is hypothesized that tau LLPS could act to initiate pathogenic tau aggregation in tauopathies, such as AD and FTD [7,19]. The pathological tau condensate’s maturation from liquid to viscous gel and finally to insoluble solid protein fibrillar aggregate gives rise to multiple protein aggregation and neurodegenerative diseases. In our study, we found that in the absence of *N*-terminal inserts, tau LLPS cannot be conducted. In the fetal human brain, tau protein does not have *N*-terminal insertions, and 2*N*-Tau interacting proteins, such as apoA1, were specifically associated with neurological disease [31]. Interestingly, an in vitro study reported that isoforms lacking *N*-terminal inserts formed small, globular oligomers that did not go on to elongate into straight filaments or paired helical filaments in buffer containing 150 μM arachidonic acid, 10 mM HEPES (pH 7.6), 100 mM NaCl, and 5 mM dithiothreitol [32].

Assembly and disassembly of condensates can serve as a sensor or switch for changes in the environment that require a homeostatic response [33]. The high mobility of tau on the shell revealed by FRAP also suggests the exchangeability of tau itself and other interactive biomolecules on this established architecture. Biomolecular liquid droplets have the ability to store molecules or concentrate chemical processes in space to serve functions in controlling the subcellular localization of specific molecules and processes [34]. PML nuclear bodies form hollow-shell structures, which helps to harbor cargos and clients in performing specific biological functions [35]. ALS-related TDP-43 forms dynamic, reversible, liquid droplet-like nuclear bodies (NBs) under stress conditions, and these TDP-43 NBs interact with LncRNA NEAT1 to alleviate cytotoxicity [36]. To better understand the structure-function relationship of this particular hollow-shell, liquid-like tau droplet, further exploration should be addressed to the determination of certain interactive biomolecules and biological processes involved in tau LLPS. The irreversible part of tau condensate shows the immobile fraction existing in the tau droplet, which might have a seeding effect on further progressive tau aggregation and the droplet for liquid-to-solid phase transition [7,28].

Taken together, *N*-terminal inserts of tau have exhibited tremendous importance in tau LLPS and progressive pathological tau aggregation. Concerning the liquid-to-solid phase transition in tauopathies [7,18], *N*-terminal inserts of tau can be considered the potential tunable therapeutic target to use when intervening in neurodegenerative disease progression at the early stages. Besides, as one of the current therapeutic approaches to tauopathies is to find highly specific inhibitors to prevent tau pathological phase transition [37], our continuing work is relatedly focused on developing an AD cell model based on the 2*N*-tau-EGFP HT22 to undergo a high-throughput screening to identify small-molecule compounds that could restore the normal behavior of tau condensates [38].

Autophagy is a lysosomal degradation pathway and plays crucial roles in health and disease [21]. The autophagy adaptor p62 phase separation drives autophagic cargo segregation with the interaction between p62 and ubiquitinated proteins [25]. In human tauopathies, the autophagy p62 co-localizes with tau inclusions [39,40,41]. Furthermore, p62 is also a versatile protein that serves as an interactive hub in multiple signaling, including those mediated by Nrf2, NF-κB, caspase-8, and mTORC1, which are involved in the delicate balance to maintain protein homeostasis and overall cell health [42,43]. The co-condensation of p62 and tau droplets may indicate consequent autophagic degradation to protect against aggregation-prone tau accumulates, which is connected to the immobile fraction of tau condensates shown in our FRAP results. Considering that the adult human brain contains these three forms of tau (2*N*, 1*N*, 0*N*), the abnormal microtubule packing and lack of colocalization with p62 condensates in 1*N*-tau and 0*N*-tau may reveal that they play roles in other distinct biochemical features. Here we addressed the biological importance of *N*-terminal insets as a regulator to promote tau condensates’ biogenesis and maturation under physiological conditions.

Additionally, we observed that when cells were fixed with 4% paraformaldehyde, the immunofluorescence observation with stably-expressed tau-EFGP in HT22 showed a more diffused fluorescence signal (Figure 2D), while the state of tau-EFGP found in living HT22 cells presented a more spatially filamentous networking structure (Figure 2C), which showed a difference in protein morphology in fixed cells and live cells, and we believe that the live-cell imaging results are closer to the real physiological state of tau protein.

In summary, the *N*-terminal inserts are connected with tau droplet biogenesis and drive tau LLPS. Our results revealed that the phase separation of tau under physiological conditions may be initialized by the intrinsically disordered *N*-terminal inserts of tau and subsequently combined with aggregation-prone *C*-terminal MTBR, which may stabilize tau condensates to undergo a liquid-to-solid phase transition [7] and to result in pathological tau aggregation and further neurofibrillary tangles in neurodegenerative diseases. Our findings provide insights into the tau LLPS assembly mechanism and the functional effects of *N*-terminal inserts in tau.

## 4. Materials and Methods

### 4.1. Recombinant Plasmids Construction for Tau Expression

pMSCV-IRES-GFP II (pMIG II) was purchased from Addgene (Watertown, MA, USA) (plasmid no. 52107). The cDNA of human tau was subcloned into the pMIG-II retroviral vector. The identities of tau variants were confirmed by nucleotide sequencing. Constructed plasmids were transfected into packaging cell line Plat-A cells with Lipo8000 (C0533, Beyotime, Shanghai, China) for the production of the retrovirus. The viral supernatant was collected to transduce HT22 cells with 10 μg/mL polybrene (C0351, Beyotime, Shanghai, China) to construct stably-expressed 2*N*, 1*N*, and 0*N*-tau-EGFP in HT22 cells.

### 4.2. Cell Cultures

Plat-A cells were purchased from Cyagen Biosciences (Santa Clara, CA, USA) (HEKPA-30001). Murine hippocampal neuronal cell line HT22 was purchased from Biofeng, Shanghai, China (SCC129). Cells were maintained in Dulbecco’s modified Eagle’s medium (DMEM) (Hyclone, Waltham, MA, USA) supplemented with 10% fetal bovine serum (FBS) (10099-141C, Gibco, Waltham, MA, USA), 100 μg/mL streptomycin, and 100 units/mL penicillin (15140-122, Gibco, Waltham, MA, USA) in a humidified incubator with 5% CO_2_.

### 4.3. Live Cell Imaging and Fluorescence Recovery after Photobleaching (FRAP)

HT22 cells were seeded in FluorDish (FD35-100, World Precision Instruments, Sarasota, FL, USA) the day before the experiment to let cells attach to the cover glass bottom. One hour before the experiment, the culture medium was changed to DMEM without phenol red (21063-029, Gibco, Waltham, MA, USA). Live-cell imaging and real-time tracking of cell dynamics were acquired with a Carl Zeiss LSM710 laser scanning confocal microscope (Carl Zeiss, Jena, Germany) with a 63× oil immersion (1.4 NA) objective in a Z-stack. The microscope was equipped with a live-cell imaging system module at 5% CO_2_ and 37 °C to maintain the cell cultivation environment during the experiment.

The FRAP experiment was performed on stably-expressed tau-EGFP droplets [7] in HT22 live cells using the 488 nm laser line with Carl Zeiss LSM710. Each droplet was selected inside a round circle defined as ROI (region of interest) and bleached at 100% laser power. For each time-lapse image, the mean fluorescence intensities of the same three regions were recorded (ROI1 = photobleached region; ROI2 = unbleached region in the same droplet to correct for the overall photobleaching; and ROI3 = background signal outside of the droplet was subtracted from both ROI1 and ROI2 intensities). Reference ROIs were defined in the cytosol of the same cell (ROI2) and outside of the cell (ROI3). The recovery time constant was plotted with the corrected fluorescence intensities versus time using GraphPad Prism 8.0 [7]. Images were analyzed with Image J (NIH, Bethesda, MD, USA).

### 4.4. Immunofluorescence and Microscopy

Cells were seeded on the coverslip in a 6-well plate at 6 × 10^5^ cells per well the day before the experiment. On the next day, cells were fixed in 4% paraformaldehyde for 15 min, then washed with PBS. Cells were mounted in antifade mounting medium vectashield (H-1000, Vector Laboratories, Burlingame, CA, USA). Immunofluorescence images were acquired with a Carl Zeiss LSM710 laser scanning confocal microscope using a 63× oil immersion objective in a Z-stack. The primary antibody used for p62/SQSTM1 was pAb (18420-1-AP, Proteintech, Rosemont, IL, USA, diluted in 1:1000) with overnight incubation at 4 °C. The secondary antibody (Alexa Fluor 647, A0468, Beyotime, Shanghai, China, diluted in 1:1000) was applied for 2 h at room temperature. After washing 3 times with 0.1% TBST, it was mounted with vectashield. Images were analyzed with Image J (NIH, Bethesda, MD, USA).

### 4.5. Protein Extraction and Western Blotting

The stably-transfected HT22 cells were seeded in a 6-well plate at 3 × 10^5^ cells per well the day before the experiment. After cultivation of 24 h, total cell lysates were scraped and collected by directly adding boiling sample loading buffer. Then the cell lysates were sonicated (SCIENTZ-II D, Ningbo, China) to shear sticky DNA. Proteins were separated by 10% SDS-PAGE (EpiZyme, Shanghai, China) and transferred onto nitrocellulose membranes (Whatman, Maidstone, UK). The expression of tau variants was detected with primary antibody HT7 (MN1000, ThermoFisher, Waltham, MA, USA, diluted in 1:2000) by overnight incubation at 4 °C. The beta-actin mAb (5B7, YM3028, Immunoway, Plano, TX, USA) was used as the internal control. The secondary antibodies (Anti-mouse IgG, HRP-linked antibody; Anti-rabbit IgG, HRP-linked antibody, Cell Signaling, Danvers, MA, USA, diluted in 1:2000) were applied for 1 h at room temperature. The membranes were developed using the ECL detection system.

### 4.6. Disorder Prediction and Inter-Protein Contact Prediction

The disorder of human full-length tau (2N4R, 441 amino acids) was predicted using PONDR (Predictor of Natural Disordered Regions) [44]. Interfacial residue–residue contact prediction between tau and p62 was predicted using ComplexContact [45]. The tau amino acid sequence from Uniprot (P10636-8) and p62 amino acid sequence from Uniprot (Q13501-1) were used.

## References

[1] Hyman A., Weber C.A., Julicher F. (2014). Liquid-Liquid Phase Separation in Biology. Annu. Rev. Cell Dev. Biol..

[2] Lafontaine D.L.J., Riback J.A., Bascetin R., Brangwynne C.P. (2020). The nucleolus as a multiphase liquid condensate. Nat. Rev. Mol. Cell Biol..

[3] Banani S.F., Lee H.O., Hyman A.A., Rosen M.K. (2017). Biomolecular condensates: Organizers of cellular biochemistry. Nat. Rev. Mol. Cell. Biol..

[4] Shin Y., Brangwynne C.P. (2017). Liquid phase condensation in cell physiology and disease. Science.

[5] Molliex A., Temirov J., Lee J., Coughlin M., Kanagaraj A.P., Kim H.J., Mittag T., Taylor J.P. (2015). Phase Separation by Low Complexity Domains Promotes Stress Granule Assembly and Drives Pathological Fibrillization. Cell.

[6] Uversky V.N. (2017). Intrinsically disordered proteins in overcrowded milieu: Membrane-less organelles, phase separation, and intrinsic disorder. Curr. Opin. Struct. Biol..

[7] Wegmann S., Eftekharzadeh B., Tepper K., Zoltowska K.M., Bennett R.E., Dujardin S., Laskowski P.R., MacKenzie D.M., Kamath T., Commins C. (2018). Tau protein liquid–liquid phase separation can initiate tau aggregation. EMBO J..

[8] Lin Y., Fichou Y., Zeng Z., Hu N.Y., Han S. (2020). Electrostatically Driven Complex Coacervation and Amyloid Ag-gregation of Tau Are Independent Processes with Overlapping Conditions. ACS Chem. Neurosci..

[9] Orr M.E., Sullivan A.C., Frost B. (2017). A Brief Overview of Tauopathy: Causes, Consequences, and Therapeutic Strategies. Trends Pharmacol. Sci..

[10] Avila J., Lucas J.J., Pérez M., Hernández F. (2004). Role of Tau Protein in Both Physiological and Pathological Conditions. Physiol. Rev..

[11] Ait-Bouziad N., Lv G., Mahul-Mellier A.-L., Xiao S., Zorludemir G., Eliezer D., Walz T., Lashuel H.A. (2017). Discovery and characterization of stable and toxic Tau/phospholipid oligomeric complexes. Nat. Commun..

[12] Boyko S., Qi X., Chen T.-H., Surewicz K., Surewicz W.K. (2019). Liquid–liquid phase separation of tau protein: The crucial role of electrostatic interactions. J. Biol. Chem..

[13] Ambadipudi S., Biernat J., Riedel D., Mandelkow E., Zweckstetter M. (2017). Liquid–liquid phase separation of the microtubule-binding repeats of the Alzheimer-related protein Tau. Nat. Commun..

[14] Ferreon J.C., Jain A., Choi K.-J., Tsoi P.S., MacKenzie K.R., Jung S.Y., Ferreon A.C. (2018). Acetylation Disfavors Tau Phase Separation. Int. J. Mol. Sci..

[15] Ash P.E.A., Lei S., Shattuck J., Boudeau S., Carlomagno Y., Medalla M., Mashimo B.L., Socorro G., Al-Mohanna L.F.A., Jiang L. (2021). TIA1 potentiates tau phase separation and promotes generation of toxic oligomeric tau. Proc. Natl. Acad. Sci. USA.

[16] Ambadipudi S., Reddy J.G., Biernat J., Mandelkow E., Zweckstetter M. (2019). Residue-specific identification of phase separation hot spots of Alzheimer’s-related protein tau. Chem. Sci..

[17] Zhang X., Vigers M., McCarty J., Rauch J.N., Fredrickson G.H., Wilson M.Z., Shea J.-E., Han S., Kosik K.S. (2020). The proline-rich domain promotes Tau liquid–liquid phase separation in cells. J. Cell Biol..

[18] Boyko S., Surewicz K., Surewicz W.K. (2020). Regulatory mechanisms of tau protein fibrillation under the conditions of liquid–liquid phase separation. Proc. Natl. Acad. Sci. USA.

[19] Kanaan N.M., Hamel C., Grabinski T., Combs B. (2020). Liquid-liquid phase separation induces pathogenic tau con-formations in vitro. Nat. Commun..

[20] McSwiggen D.T., Mir M., Darzacq X., Tjian R. (2019). Evaluating phase separation in live cells: Diagnosis, caveats, and functional consequences. Genes Dev..

[21] Aparicio R., Rana A., Walker D.W. (2019). Upregulation of the Autophagy Adaptor p62/SQSTM1 Prolongs Health and Lifespan in Middle-Aged Drosophila. Cell Rep..

[22] Wheeler J.R., Matheny T., Jain S., Abrisch R., Parker R. (2016). Distinct stages in stress granule assembly and disassembly. eLife.

[23] Feric M., Vaidya N., Harmon T.S., Mitrea D.M., Zhu L., Richardson T.M., Kriwacki R.W., Pappu R.V., Brangwynne C.P. (2016). Coexisting Liquid Phases Underlie Nucleolar Subcompartments. Cell.

[24] Danieli A., Martens S. (2018). p62-mediated phase separation at the intersection of the ubiquitin-proteasome system and autophagy. J. Cell Sci..

[25] Sun D., Wu R., Zheng J., Li P., Yu L. (2018). Polyubiquitin chain-induced p62 phase separation drives autophagic cargo segregation. Cell Res..

[26] Musi N., Valentine J.M., Sickora K.R., Baeuerle E., Thompson C.S., Shen Q., Orr M.E. (2018). Tau protein aggregation is associated with cellular senescence in the brain. Aging Cell.

[27] Brunello C.A., Merezhko M., Uronen-Mattila R.-L., Huttunen H.J. (2019). Mechanisms of secretion and spreading of pathological tau protein. Cell. Mol. Life Sci..

[28] Zbinden A., Pérez-Berlanga M., De Rossi P., Polymenidou M. (2020). Phase Separation and Neurodegenerative Diseases: A Disturbance in the Force. Dev. Cell.

[29] Takuma H., Arawaka S., Mori H. (2003). Isoforms changes of tau protein during development in various species. Dev. Brain Res..

[30] Dujardin S., Bégard S., Caillierez R., Lachaud C., Carrier S., Lieger S., Gonzalez J.A., Deramecourt V., Déglon N., Maurage C.-A. (2018). Different tau species lead to heterogeneous tau pathology propagation and misfolding. Acta Neuropathol. Commun..

[31] Liu C., Song X., Nisbet R., Götz J. (2016). Co-immunoprecipitation with Tau Isoform-specific Antibodies Reveals Distinct Protein Interactions and Highlights a Putative Role for 2N Tau in Disease. J. Biol. Chem..

[32] King M.E., Gamblin T.C., Kuret J., Binder L.I. (2002). Differential Assembly of Human Tau Isoforms in the Presence of Arachidonic Acid. J. Neurochem..

[33] Lyon A.S., Peeples W.B., Rosen M.K. (2020). A framework for understanding the functions of biomolecular condensates across scales. Nat. Rev. Mol. Cell Biol..

[34] Bergeron-Sandoval L.-P., Safaee N., Michnick S.W. (2016). Mechanisms and Consequences of Macromolecular Phase Separation. Cell.

[35] Lallemand-Breitenbach V., de The H. (2018). PML nuclear bodies: From architecture to function. Curr. Opin. Cell. Biol..

[36] Wang C., Duan Y., Duan G., Wang Q., Zhang K., Deng X., Qian B., Gu J., Ma Z., Zhang S. (2020). Stress Induces Dynamic, Cytotoxicity-Antagonizing TDP-43 Nuclear Bodies via Paraspeckle LncRNA NEAT1-Mediated Liquid-Liquid Phase Separation. Mol. Cell.

[37] Seidler P.M., Boyer D.R., Rodriguez J.A., Sawaya M.R., Cascio D., Murray K., Gonen T., Eisenberg D.S. (2017). Structure-based inhibitors of tau aggregation. Nat. Chem..

[38] Xiao S., Lu Y., Wu Q., Yang J., Chen J., Zhong S., Eliezer D., Tan Q., Wu C. (2021). Fisetin inhibits tau aggregation by interacting with the protein and preventing the formation of β-strands. Int. J. Biol. Macromol..

[39] Falcon B., Noad J., McMahon H., Randow F., Goedert M. (2018). Galectin-8–mediated selective autophagy protects against seeded tau aggregation. J. Biol. Chem..

[40] Kuusisto E., Salminen A., Alafuzoff I. (2001). Ubiquitin-binding protein p62 is present in neuronal and glial inclusions in human tauopathies and synucleinopathies. NeuroReport.

[41] Terni B., Rey M.J., Boluda S., Torrejón-Escribano B., Sabate M.P., Calopa M., Van Leeuwen F.W., Ferrer I., Calopa M. (2007). Mutant ubiquitin and p62 immunoreactivity in cases of combined multiple system atrophy and Alzheimer’s disease. Acta Neuropathol..

[42] Emanuele S., Lauricella M., D’Anneo A., Carlisi D., De Blasio A., Di Liberto D., Giuliano M. (2020). p62: Friend or Foe? Evidences for OncoJanus and NeuroJanus Roles. Int. J. Mol. Sci..

[43] Komatsu M., Kageyama S., Ichimura Y. (2012). p62/SQSTM1/A170: Physiology and pathology. Pharmacol. Res..

[44] http://www.pondr.com/.

[45] Zeng H., Wang S., Zhou T., Zhao F., Li X., Wu Q., Xu J. (2018). ComplexContact: A web server for inter-protein contact prediction using deep learning. Nucleic Acids Res..

